# Meeting Needs of Diverse Older Adults and Caregivers During Public Health Emergencies: What Can We Learn From COVID-19?

**DOI:** 10.1093/geroni/igab046.893

**Published:** 2021-12-17

**Authors:** Alycia Bayne, Rachel Singer, Candace Spradley, Lauren Isaacs, Eva Jeffers, Benjamin Olivari, Kevin Matthews, Alaina Whitton

**Affiliations:** 1 NORC at the University of Chicago, Bethesda, Maryland, United States; 2 CDC Foundation, Atlanta, Georgia, United States; 3 NORC at the University of Chicago, Chicago, Illinois, United States; 4 Centers for Disease Control and Prevention (CDC), Atlanta, Georgia, United States; 5 NCCDPHP, Centers for Disease Control, Georgia, United States; 6 The CDC Foundation, Atlanta, Georgia, United States

## Abstract

With support from the CDC Foundation and technical assistance from the Centers for Disease Control and Prevention, NORC at the University of Chicago conducted studies to examine the needs and concerns of older adults and unpaid caregivers during COVID-19, including their trusted sources of COVID-19 information and available public health interventions. Methods included a nationally representative survey of 1,030 adults aged 50+ years using computer-assisted telephone and web interviewing; online focus groups with older adults and caregivers in Spanish and English; a survey and interviews with stakeholder organizations; secondary analysis of U.S. caregiver surveys; analysis of public social media posts; and searches of peer-reviewed and grey literature in Spanish and English to identify interventions. Results suggest that needs and concerns differed among older adult subpopulations, including racial and ethnic minority populations, people with lower incomes, rural and tribal populations, people with limited English proficiency, and people with disabilities as well as caregivers. Older adults perceived news media, the internet, and healthcare providers as important resources for COVID-19 information, although trusted sources varied by race and ethnicity, urbanicity, and income. Findings suggested the need to increase awareness of existing public health interventions and resources to support older adults and caregivers during public health emergencies like COVID-19. Strategies for tailoring communication for diverse older adults and caregivers include partnering with national organizations, leveraging community-level infrastructure, and disseminating information through trusted sources. Studying the needs of older adults and caregivers during COVID-19 can inform future public health emergency response priorities.

